# The Aging Kidney—As Influenced by Heavy Metal Exposure and Selenium Supplementation

**DOI:** 10.3390/biom11081078

**Published:** 2021-07-22

**Authors:** Jan Aaseth, Jan Alexander, Urban Alehagen, Alexey Tinkov, Anatoly Skalny, Anders Larsson, Guido Crisponi, Valeria Marina Nurchi

**Affiliations:** 1 Research Department, Innlandet Hospital, P.O. Box 104, N-2381 Brumunddal, Norway; 2 Faculty of Health and Social Sciences, Inland Norway University of Applied Sciences, N-2624 Lillehammer, Norway; 3 Norwegian Institute of Public Health, P.O. Box 222, N-0213 Oslo, Norway; jan.alexander@fhi.no; 4 Division of Cardiovascular Medicine, Department of Medical and Health Sciences, Linköping University, SE-581 85 Linköping, Sweden; urban.alehagen@liu.se; 5 Laboratory of Biotechnology and Bioelementology, Yaroslavl State University, Sovetskaya Str. 14, 150000 Yaroslavl, Russia; a.a.tinkov@gmail.com; 6 IM Sechenov First Moscow State Medical University (Sechenov University), Bolshaya Pirogovskaya St., 2-4, 119146 Moscow, Russia; skalny3@microelements.ru; 7 K.G. Razumovsky Moscow State University of Technologies and Management, Zemlyanoi Val St., 73, 109004 Moscow, Russia; 8 Department of Medical Sciences, Uppsala University, SE-751 85 Uppsala, Sweden; anders.larsson@akademiska.se; 9 Department of Life and Environmental Sciences, University of Cagliari, Cittadella Universitaria, 09042 Monserrato-Cagliari, Italy; crisponi@unica.it

**Keywords:** renal disease, aging, mercury, cadmium, lead, thiols, selenium

## Abstract

The aging process in the kidneys has been well studied. It is known that the glomerular filtration rate (GFR) declines with age in subjects older than 50–60 years. However, there is still insufficient knowledge regarding the response of the aged kidney to environmental toxicants such as mercury, cadmium, and lead. Here, we present a review on the functional decline and proposed mechanisms in the aging kidney as influenced by metal pollutants. Due to the prevalence of these toxicants in the environment, human exposure is nearly unavoidable. Further, it is well known that acute and chronic exposures to toxic metals may be detrimental to kidneys of normal adults, thus it may be hypothesized that exposure of individuals with reduced GFR will result in additional reductions in renal function. Individuals with compromised renal function, either from aging or from a combination of aging and disease, may be particularly susceptible to environmental toxicants. The available data appear to show an association between exposure to mercury, cadmium and/or lead and an increase in incidence and severity of renal disease in elderly individuals. Furthermore, some physiological thiols, as well as adequate selenium status, appear to exert a protective action. Further studies providing improved insight into the mechanisms by which nephrotoxic metals are handled by aging kidneys, as well as possibilities of therapeutic protection, are of utmost importance.

## 1. Introduction

The kidney appears to be a major site of age-related changes, in addition to being a target for many environmental pollutants [1]. Long-term exposure to heavy metals such as mercury, lead, and cadmium may accelerate age-related renal deteriorations, which in part can be ascribed to the tendency of the accumulation of heavy metals in the kidneys during the processing of primary urine. Due to the increased life expectancy of humans living in the modern world, together with an increasing level of environmental metal pollutants with long elimination half-lives, it is likely that older individuals today accumulate higher levels of such toxic agents than individuals did some decades ago. Furthermore, the number of older individuals is increasing. Globally, more than 10% of the population are over the age of 60, and this percentage is predicated to rise substantially by 2050 [2]. A thorough understanding of the impact of age on various organs, including on the kidneys, is crucial when managing general healthcare, since elderly individuals make up a significant fraction of healthcare patients.

Numerous physiological changes occur in the aging kidneys, especially after the age of 70. Although healthy elderly individuals appear to be capable of maintaining normal renal function in spite of significant structural and physiological changes, this is achieved at the cost of the renal functional reserve. However, when the functional reserve is lost, kidneys have a reduced capacity to respond to external challenges, involving reduced ability to eliminate toxicants. Thus, old individuals may be more susceptible than younger ones when exposed to toxic metals from the environment.

The aging process results in numerous changes at the cellular and molecular levels. One of these changes involves a decreased ability to repair injured cells [3]. Concomitantly, acute phase reactants such as, e.g., C-reactive protein (CRP), tumor necrosis factor alpha (TNF-α), and interleukin-6 (IL-6) are expressed at higher levels [4].

Mitochondrial injuries appear to be an important factor in cellular senescence. The free radical theory of aging [5] states that generation and leakage of ROS (reactive oxygen species) from the mitochondrial respiratory chain increases with age and leads to intracellular oxidative damage. Deterioration of mitochondrial DNA will impair the function of the respiratory chain, which is accompanied by additional ROS formation and DNA injuries. These events are hypothesized to involve a continuous cycle of reactive radical formation that may lead to accelerated aging [6]. Several studies have indicated that aging is related to a declining expression of various anti-oxidative stress-related enzymes such as the superoxide dismutases (SOD1 and SOD2), catalase, and the glutathione peroxidases (GPXs) [7]. A reduction in the activities of these protective enzymes may lead to a further increase in oxidative stress and cellular aging. Exposure to mercury, cadmium, or lead, even on a low-grade scale, is known to affect anti-oxidative enzyme systems [8,9] and may thus promote age-dependent organ changes, especially in the kidneys [10]. The aim of the present review is to discuss the renal toxicity of mercury, cadmium, and lead compounds in elderly subjects, and the possible protective role of sulfur and selenium compounds.

## 2. Mercury, Cadmium and Lead—Nephrotoxic Environmental Pollutants

Toxic metals are abundant in the general environment, and at even higher levels in some occupational settings, implying that human exposure to these metals is inevitable. The cumulated exposure in elderly individuals to these nephrotoxic pollutants may promote age-dependent progression of renal deterioration [11]. Due to their function as the major route of excretion from the body, kidneys in aged individuals are especially vulnerable to heavy metal toxicity [10], mostly to mercury (Hg), cadmium (Cd), and lead (Pb). As for mercury, even minor exposures from its use in dental amalgams, vaccines, eye drops, and in traditional folk medicines may give rise to nephrotoxic effects, which may be difficult to assess because effects usually arise months or years after a low or moderate exposure [12,13]. Mercury is known to significantly affect human biochemical processes by interfering with the complex redox machinery used to regulate cell survival and mitochondrial function [14]. Cells with increased oxidative stress, for instance due to an inflammatory reaction in an aged individual, are presumed to be more susceptible to Hg toxicity than healthy cells under controlled conditions. Mercury occurs in three main forms, viz. elemental mercury (Hg^0^), organic mercury (e.g., CH_3_Hg^+^, here denoted MeHg), and inorganic mercury (Hg^2+^, Hg^+^), the latter forms often occurring as salts (e.g., HgCl_2_) [15]. All these forms have effects on the kidneys [16]. While inorganic Hg compounds are well-known nephrotoxic agents, exposure to elemental mercury vapor or to organic mercury may also involve nephrotoxicity in addition to their neurotoxicity. Elemental mercury (Hg^0^) is a heavy liquid at room temperature; it is highly volatile and at saturation at 25 °C one m^3^ of air contains 20 mg of Hg^0^ that can be rapidly absorbed upon inhalation [17]. After uptake, a part of Hg^0^ is oxidized to the nephrotoxic Hg^2+^ form [18].

Epidemiological studies gave evidence of renal injury following not only acute but also chronic exposure to various forms of mercury [19,20]. The most severe nephropathy is induced following exposure to inorganic salts of Hg^2+^ [16,21]. Accumulation of mercury in proximal tubular cells has been found to exert negative effects on antioxidative enzymes [22]. Thus, long-term exposure to mercury has been reported to decrease renal expression of enzymes involved in protective actions such as NADPH-quinone oxidoreductase and glutathione S-transferase [23]. In experiments with healthy rats exposed to HgCl_2_, renal levels of SOD, catalase, and glutathione (GSH) were lowered, indicating the oxidative effects of Hg^2+^ [24]. Apparently, many of the injurious cellular effects of long-term mercury exposure, even at low doses, are similar to those induced by aging.

As for cadmium (Cd), severe pollution with this metal was first recognized by its skeletal manifestation named the *itai-itai* disease in Japan [25]. A few decades later, experimental studies revealed the harmful consequences of Cd^2+^ involving severe damage and histological changes in the kidneys, along with renal dysfunction [25].

In the liver and other tissues, Cd^2+^ forms a complex with the low molecular weight protein metallothionein (MT), which can be transported to and filtered by glomeruli, followed by reabsorption into the proximal tubuli. Intracellularly, in tubular cells, the MT-complex releases free Cd^2+^ upon overloading, thus causing renal damage, ia. through perturbing calcium homeostasis, inducing oxidative stress, and downregulating mitochondrial enzymes [26,27]. The Cd^2+^-induced damage to proximal tubuli, identified as a reabsorptive dysfunction, is manifested by a characteristic proteinuria that may include albumin, but otherwise is dominated by low molecular weight proteins of which β_2_-microglobulin and *N*-acetyl-β-d-glucosaminidase are used as markers [28]. A health survey in Sweden of women around 60 years of age disclosed associations between low levels of urinary Cd (around 0.6 μg/L) and increased levels of *N*-acetyl-β-d-glucosaminidase in urine, and also the effects on GFR [29]. The effects of low-level Cd exposure on renal tubular function were also observed in a later study by Wallin et al. [30]. An increased susceptibility for patients with diabetes to develop tubular dysfunction upon low to moderate Cd^2+^ exposure has been observed [31]. Associations between cadmium exposure and arterial hypertension have also been reported [32].

Regarding compounds of lead (Pb), these pollutants are usually absorbed readily by the intestines as well as by lungs upon exposure. From the circulation, Pb^2+^ is distributed into different tissues and organs, including the liver and kidneys, where it may cause oxidative damage to cells, ia. by uncoupling the respiratory chain in mitochondria [33]. Different hypotheses have been forwarded to explain the kidney toxicity of Pb^2+^. Due to ionic similarities, Pb^2+^ may dysregulate the calcium homeostasis. As a result, Ca^2+^ release from mitochondria is stimulated, accompanied by opening of the mitochondrial transitional pores, resulting in generation of reactive species and oxidative stress [34]. Among the renal cells, proximal tubuli appear to be particularly susceptible to Pb^2+^-induced damage, and studies on primary cultures of rat proximal tubular cells conformed to the assumption that Pb^2+^ elevates cytosol Ca^2+^ at the expense of mitochondrial Ca^2+^ [35]. Epidemiological associations between lead exposure and arterial hypertension have been observed [36]. In a prospective study [37] the observed decline in renal function among middle-aged and elderly individuals appeared to depend both on lead stores and circulating lead, the decline in renal function being most pronounced among the individuals with diabetes or hypertension at inclusion. Another prospective study on a cohort with age at inclusion of almost 60 years and a follow-up period of 16 years revealed that even low-level lead exposure was associated with decreased kidney function [38].

## 3. Functional Changes in Aging Kidneys and the Role of Environmental Pollutants

According to Denic et al. [39], almost 40% of the renal glomeruli become sclerotic by the eighth decade of life. The pathogenesis of glomerulosclerosis is thought to involve several factors including alterations in blood flow and increased susceptibility to inflammatory cytokines [40]. The phenomenon of increased inflammatory response in the elderly may be related to reduced expression of sirtuins [41]. Existing data indicate that exposure to Cd, Hg, and Pb can inhibit SIRT1 activity and thus exert proinflammatory actions [42]. As nephrons are lost due to aging and inflammation, compensatory alterations occur in the remaining nephrons leading to glomerular hyperfiltration and proteinuria [43].

Age-related changes also occur in renal tubuli, ia. with interstitial inflammation and fibrosis [44]. Deposition of collagens, mediated by invading cells, is involved in the pathogenesis of a slowly developing fibrosis. Structural changes are paralleled by alterations in tubular function, leading ia. to a reduced ability to concentrate urine.

It has been estimated that the glomerular filtration rate (GFR) decreases, in average, by approximately 10% per decade of life after an age of about 50–60 years [43]. This decrease has in part been ascribed to reduction in the total number of functioning nephrons [45]. Aging also affects renal blood flow, presumably reflecting changes in cardiac output and changed vascular resistance in afferent and efferent arterioles [46].

In patients with diseases such as diabetes and hypertension, the decline in renal function is usually more pronounced than in subjects without these diseases [47]. It has also become apparent that progression of renal failure, for instance due to poorly controlled diabetes, occurs more rapidly in elderly subjects compared with younger ones. Hypertension, cardiovascular disease, diabetes, or metabolic syndrome with insulin resistance, which are common in the elderly population, are considered significant risk factors for the development of overt renal failure [48]. In USA, as in Europe, about 65% of adults over the age of 60 have been diagnosed with hypertension, and a similar trend exists for diabetes [49]. Thus, together with accumulation of heavy metals and other environmental pollutants, diseases such as hypertension and diabetes may accelerate the physiological age-related decline in renal function [10].

Heavy metals are largely deposited in renal tubuli thus leading to much higher concentrations of heavy metals in tubular cells than in the rest of the body. Since heavy metals mainly cause damage to the tubular cells, a typical pattern in heavy metal poisoning is tubular proteinuria. The reabsorption and concentration of metal ions in the tubular cells is usually an energy requiring process, as they are in most cases carried by amino acid transporters. In general, an early urinary marker for tubular damage is the kidney injury molecule (KIM-1) [50]. Urinary β_2_-microglobulin (β_2_M) is regularly used to monitor kidney status and suspected injuries in industrial workers exposed to heavy metals.

A combination of two types of exposures, atherosclerosis, and heavy metals, will most likely increase the risk of injury. Kidney injuries in clinical medicine are mainly monitored by urine albumin and urine albumin/creatinine ratio, which mainly detect glomerular injuries, even if use of biomarkers for tubular injury may give important additional information.

Although urinary excretion of low-molecular weight protein is an early sign of cadmium-induced kidney damage, hypercalciuria also represents a sign of tubular dysfunction, and together with the disruption of the vitamin D metabolism can contribute to the development of osteoporosis [51].

Exposure to inorganic mercury may lead to heavy proteinuria with hypoproteinemia and edema [52]. Today, the most common route of human exposure to mercury compounds is via the ingestion of food, primarily of fish contaminated with MeHg. Large predatory fishes, such as swordfish and shark, may contain high levels of MeHg and represent a major source of mercury exposure [53]. Upon ingestion, MeHg is rapidly absorbed by the gastrointestinal tract, with some being distributed to the kidneys, mostly after biotransformation to the inorganic form [54].

Recent epidemiological studies in human populations indicate that the renal burden of mercury increases with age [55]. Interestingly, chronic exposure to MeHg has been reported to correlate with development of type II diabetes and hypertension [56]. Apparently, exposure to mercury may enhance the progression of renal failure. A study of residents living near a mine in southwestern China reported that individuals above 60 years of age had higher blood mercury and increased serum creatinine as compared with younger adults in the same area [57]. Altogether, several studies have shown that prolonged exposure to nephrotoxic metals, such as mercury, cadmium, and lead can exacerbate renal insufficiency in older individuals [58,59].

## 4. Interactions of Heavy Metals with Endogenous Thiols

**Figure 1:**
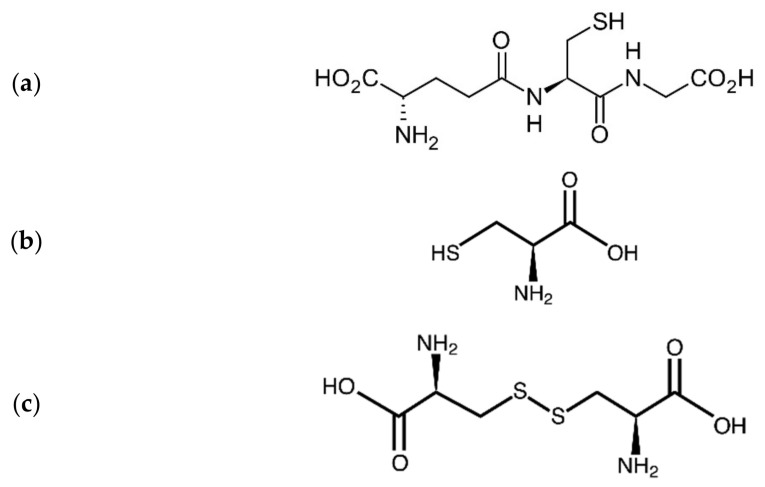
Molecular formulae of (**a**) glutathione, (**b**) cysteine and (**c**) cystine.

Within biological systems, e.g., in blood, mercury ions, and to some extent also cadmium and lead are bound to thiol-containing biomolecules, such as albumin, MT, glutathione (GSH) and cysteine (Cys-SH) [60] (Figure 1). As for renal uptake, research has indicated that mercuric ions are taken up in proximal tubular cells across the luminal border as a Cys-S-conjugate [61]. Since the conjugate Cys-S-Hg-S-Cys has similarities with the amino acid cystine (Cys-S-S-Cys) (Figure 1), it seems reasonable that this amino acid-mercuric conjugate uses the cystine transporter to enter into the tubular cells. Similarly, due to mimicry with methionine, the Cys-S-conjugates of MeHg have also been presumed to be substrates for the corresponding amino acid carrier [62]. In contrast, cadmium is considered to be taken up into the same tubular cells as complexes with the low molecular weight protein MT, after which Cd-MT complexes are transferred to lysosomes and degraded [63]. Intracellularly, MT ties up a significant part of mercuric ions in a complex that is not easily transported out of cells, leading to intracellular retention of mercuric ions, in addition to retention of other heavy metal ions [64].

Heavy metal ions, in particular mercuric ions, also have a strong affinity for GSH and may be bound and detoxified by GSH intracellularly [17]. Physiologically, the concentration of GSH in renal tubular cells is about 3 mmol/L, which makes this peptide well suited for tying up intracellular metal ions. Exposure of experimental animals to HgCl_2_ lowered renal levels of intracellular GSH [65], suggesting that GSH is utilized as a complexing and/or protecting agent during the exposure. Although the binding of heavy metal ions to intracellular SH-molecules represents a protective mechanism, the same binding may also contribute to intracellular retention of the metals.

**Figure 2:**
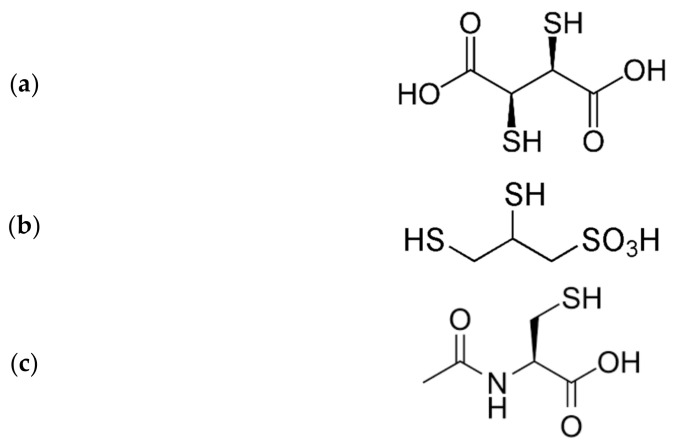
Molecular formulae of (**a**) DMSA, (**b**) DMPS and (**c**) acetylcysteine.

In chronic low-dosed exposure, acetylcysteine (Figure 2) may be used as a protecting agent due to its ability to increase the cellular GSH-levels [66], which secondarily will raise the enzymatic activity of GPX [67]. As for the chelating thiols 2,3-dimercaptopropane-1-sulfonic acid (DMPS) and 2,3-dimercaptosuccinic acid (DMSA) (Figure 2), these drugs are reserved for acute poisoning cases [68].

## 5. Selenium—A Renal Protector with Chelating Properties

The process of aging appears to be related to a redox imbalance in cells characterized by increased ROS production or decreased efficacy of ROS scavenging, resulting in impaired cellular functions [5]. Supplementation of selenium in vivo has been reported to enhance antioxidant capacity, especially by increasing antioxidant enzyme activity, e.g., the activity of GPX [69]. Of particular interest is the observed increase in serum GPX3 upon selenium supplementation, as this selenoenzyme is formed in the kidneys and found accumulated in the basement membrane surrounding renal proximal tubules [70].

A recent placebo-controlled study of an elderly Swedish population showed an association between low selenium (Se) status and age-related reduction in renal function [71]. In this study, dietary supplementation for four years with selenium 200 μg/day (as SelenoPrecise, Pharma Nord, Denmark) and coenzyme Q_10_, significantly improved kidney function as compared with the functional indices in the placebo group. The improvement of kidney function was attributed to optimized function of antioxidative selenoenzymes such as GPXs and thioredoxin reductase, although it is known that selenol compounds can also act as strong chelating agents, e.g., against mercurials [72]. However, it should be noted that supra-nutritional intakes of selenium above about 300 μg/day may exert prooxidative effects [73], and have been associated with increased risk of type 2 diabetes mellitus [74]. Interestingly, low serum selenium is commonly reported in patients with advanced renal disease [75]. Low serum selenium levels in patients on hemodialysis or peritoneal dialysis has been ascribed to diminished selenium retention due to chronic oxidative stress [76]. In a recent study on a cohort with end-stage renal disease, patients with low serum selenium values (<63 μg/L) showed an increased mortality risk, as compared to patients with normal or high selenium (>118 μg/L) [77]. One important pathway of selenium to the kidney is the uptake of circulating selenoprotein-P fragments by megalin/LRP2 a multiligand receptor mediating endocytosis in the plasma membrane of the tubular cells [78,79]. This receptor, either alone or in concert with cubulin, functions as a receptor for reabsorption from primary urine of low molecular proteins, e.g., vitamin D binding protein [80], a function that may be compromised in tubular injury.

**Figure 3:**
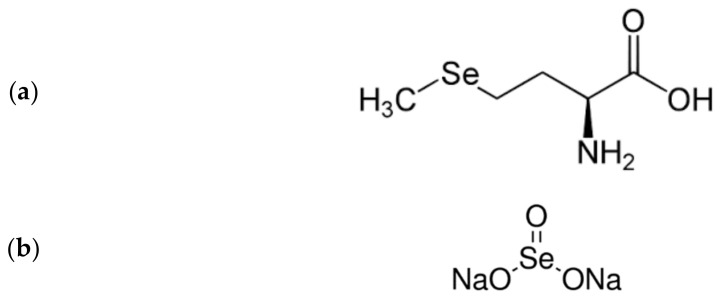
Molecular formulae of (**a**) selenomethionine and (**b**) sodium selenite.

Mercury, as well as lead and cadmium, may be bound and detoxified by selenium compounds, mainly selenite or selenomethionine (Figure 3) [72].

Several studies in humans have shown that administration of Se to individuals exposed to mercury reduced the severity of Hg intoxication [81]. However, it is not clear whether Hg-Se complexes are excretable forms of mercury. Of note, the binding affinity of mercury is greater for Se compounds than for thiols [15]. Sugiura et al. [82], from their NMR measurements, reported that the order of binding affinity of various selenium and sulfur donor groups toward methylmercury is in the order SeH > SH > Se-Se > S-S, SeCH_3_, SCH_3_. However, the concentration of selenium in blood is only about 1 µmol/L [83] while the concentration of albumin-SH, Cys, and GSH in blood is approximately 500, 275, and 850 µmol/L, respectively [84]. Since the normal blood SH-concentrations of albumin-SH and GSH (totally above1000 µmol/L) are significantly greater than that of selenium, it appears reasonable that the major fraction of circulating mercury is bound to albumin and/or GSH rather than to Se-proteins, although a minor fraction of circulating mercury is coordinated to selenium compounds. A recent review of Spiller et al. [85] remarks, besides the role of selenium supplementation, the pros and cons of chelation, and the impact of chelation and selenium on the different forms of mercury.

## 6. Discussion and Conclusions

The aging process in the kidneys has been studied and characterized comprehensively. It is well known that glomerulosclerosis leads to decreased GFR. However, there is little information regarding the response of the aged kidneys to environmental toxicants such as mercury, cadmium, and lead. Due to the prevalence of mercury, cadmium, and lead in the environment, human exposure is practically unavoidable. Further, it is well known that not only acute but also chronic exposures to toxic metals may be detrimental to the kidneys of healthy adults. Available research indicates that long-term exposure of individuals with reduced GFR to these metals may result in additional reductions in renal function. Individuals with compromised renal function, either from aging, disease, or a combination of both, may be particularly susceptible to these toxicants. Available data show an association between exposure to mercury, cadmium, and lead and an increase in incidence and severity of renal disease. Of note, early signs of renal dysfunction often go unnoticed, which implies that individuals with reduced renal function are unaware that they may be at risk. Preliminary observations indicate that some physiological thiol amino acids, as well as adequate or supra-nutritional selenium supplementation, exert nephroprotective actions, but further studies are necessary on these therapeutic possibilities. Improved insights into the manner in which heavy metals are handled by aging kidneys is of utmost importance.

## Data Availability

Not applicable.

## References

[1] Xu X., Nie S., Ding H., Hou F.F. (2018). Environmental pollution and kidney diseases. Nat. Rev. Nephrol..

[2] Pigott C.A. (2002). World Population Ageing, 1950–2050.

[3] Schmitt R., Cantley L.G. (2008). The impact of aging on kidney repair. Am. J. Physiol. Renal. Physiol..

[4] O’Brown Z.K., Van Nostrand E.L., Higgins J.P., Kim S.K. (2015). The inflammatory transcription factors NFkappaB, STAT1 and STAT3 drive age-associated transcriptional changes in the human kidney. PLoS Genet..

[5] Liochev S.I. (2013). Reactive oxygen species and the free radical theory of aging. Free Radic. Biol. Med..

[6] Poulose N., Raju R. (2014). Aging and injury: Alterations in cellular energetics and organ function. Aging Dis..

[7] Lim J.H., Kim E.N., Kim M.Y., Chung S., Shin S.J., Kim H.W., Yang C.W., Kim Y.S., Chang Y.S., Park C.W. (2012). Age-associated molecular changes in the kidney in aged mice. Oxid. Med. Cell. Longev..

[8] Teixeira F.B., de Oliveira A.C., Leão L.K., Fagundes N.C., Fernandes R.M., Fernandes L.M., Crespo-Lopez M.E. (2018). Exposure to inorganic mercury causes oxidative stress, cell death, and functional deficits in the motor cortex. Front. Mol. Neurosci..

[9] Bjørklund G., Aaseth J., Crisponi G., Rahman M.M., Chirumbolo S. (2019). Insights on alpha lipoic and dihydrolipoic acids as promising scavengers of oxidative stress and possible chelators in mercury toxicology. J. Inorg. Biochem..

[10] Bridges C.C., Zalups R.K. (2017). The aging kidney and the nephrotoxic effects of mercury. J. Toxicol. Environ. Health.

[11] Moriguchi J., Ezaki T., Tsukahara T., Fukui Y., Ukai H., Okamoto S., Shimbo S., Sakurai H., Ikeda M. (2005). Effects of aging on cadmium and tubular dysfunction markers in urine from adult women in non-polluted areas. Int. Arch. Occup. Environ. Health.

[12] Bjørklund G., Lindh U., Aaseth J., Mutter J., Chirumbolo S. (2019). Mercury in dental amalgams: A great concern for clinical toxicology in developing countries. J. Trace Elem. Med. Biol..

[13] Ye B.J., Kim B.G., Jeon M.J., Kim S.Y., Kim H.C., Jang T.W., Hong Y.S. (2016). Evaluation of mercury exposure level, clinical diagnosis and treatment for mercury intoxication. Ann. Occup. Environ. Med..

[14] Farina M., Avila D.S., Da Rocha J.B.T., Aschner M. (2013). Metals, oxidative stress and neurodegeneration: A focus on iron, manganese and mercury. Neurochem. Int..

[15] Syversen T., Kaur P. (2012). The toxicology of mercury and its compounds. J. Trace Elem. Med. Biol..

[16] Clarkson T.W. (1997). The toxicology of mercury. Crit. Rev. Clin. Lab. Sci..

[17] Bjørklund G., Crisponi G., Nurchi V.M., Cappai R., Djordjevic A.B., Aaseth J. (2019). A review on coordination properties of thiol-containing chelating agents towards mercury, cadmium, and lead. Molecules.

[18] Eide I., Syversen T.L. (1983). Relationship between catalase activity and uptake of elemental mercury by rat brain. Acta Pharmacol. Toxicol..

[19] Ha E., Basu N., Bose-O’Reilly S., Dórea J.G., McSorley E., Sakamoto M., Chan H.M. (2017). Current progress on understanding the impact of mercury on human health. Environ. Res..

[20] Pollack A.Z., Mumford S.L., Mendola P., Perkins N.J., Rotman Y., Wactawski-Wende J., Schisterman E.F. (2015). Kidney biomarkers associated with blood lead, mercury, and cadmium in premenopausal women: A prospective cohort study. J. Toxicol. Environ. Health Sci..

[21] Crisponi G., Nurchi V.M. (2015). Metal Ion Toxicity. Encyclopedia of Inorganic and Bioinorganic Chemistry.

[22] Joshi D., Kumar M.D., Kumar S.A., Sangeeta S. (2014). Reversal of methylmercury-induced oxidative stress, lipid peroxidation, and DNA damage by the treatment of N-acetyl cysteine: A protective approach. J. Environ. Pathol. Toxicol. Oncol..

[23] Al Bakheet S.A., Attafi I.M., Maayah Z.H., Abd-Allah A.R., Asiri Y.A., Korashy H.M. (2013). Effect of long-term human exposure to environmental heavy metals on the expression of detoxification and DNA repair genes. Environ. Pollut..

[24] Agrawal S., Flora G., Bhatnagar P., Flora S. (2014). Comparative oxidative stress, metallothionein induction and organ toxicity following chronic exposure to arsenic, lead and mercury in rats. Cell. Mol. Biol..

[25] Nordberg G.F. (2009). Historical perspectives on cadmium toxicology. Toxicol. Appl. Pharmacol..

[26] Eybl V., Kotyzova D., Koutensky J. (2006). Comparative study of natural antioxidants—curcumin, resveratrol and melatonin—in cadmium-induced oxidative damage in mice. Toxicology.

[27] Dua T.K., Dewanjee S., Khanra R., Bhattacharya N., Bhaskar B., Zia-Ul-Haq M., De Feo V. (2015). The effects of two common edible herbs, *Ipomoea aquatica* and *Enhydra fluctuans*, on cadmium-induced pathophysiology: A focus on oxidative defence and anti-apoptotic mechanism. J. Transl. Med..

[28] Liang Y., Lei L., Nilsson J., Li H., Nordberg M., Bernard A., Jin T. (2012). Renal function after reduction in cadmium exposure: An 8-year follow-up of residents in cadmium-polluted areas. Environ. Health Perspect..

[29] Åkesson A., Lundh T., Vahter M., Bjellerup P., Lidfeldt J., Nerbrand C., Samsioe G., Strömberg U., Skerfving S. (2005). Tubular and glomerular kidney effects in Swedish women with low environmental cadmium exposure. Environ. Health Perspect..

[30] Wallin M., Sallsten G., Lundh T., Barregard L. (2014). Low-level cadmium exposure and effects on kidney function. Occup. Environ. Med..

[31] Chen L., Lei L., Jin T., Nordberg M., Gunnar F., Nordberg M.D. (2006). Plasma metallothionein antibody, urinary cadmium, and renal dysfunction in a Chinese type 2 diabetic population. Diabetes Care.

[32] An H.C., Sung J.H., Lee J., Sim C.S., Kim S.H., Kim Y. (2017). The association between cadmium and lead exposure and blood pressure among workers of a smelting industry: A cross-sectional study. Ann. Occup. Environ. Med..

[33] Reyes J.L., Molina-Jijón E., Rodríguez-Muñoz R., Bautista-García P., Debray-García Y., Namorado M.D.C. (2013). Tight junction proteins and oxidative stress in heavy metals-induced nephrotoxicity. BioMed Res. Int..

[34] Ponce-Canchihuamán J.C., Pérez-Méndez O., Hernández-Muñoz R., Torres-Durán P.V., Juárez-Oropeza M.A. (2010). Protective effects of Spirulina maxima on hyperlipidemia and oxidative-stress induced by lead acetate in the liver and kidney. Lipids Health Dis..

[35] Wang H., Wang Z.-K., Jiao P., Zhou X.-P., Yang D.-B., Wang Z.-Y., Wang L. (2015). Redistribution of subcellular calcium and its effect on apoptosis in primary cultures of rat proximal tubular cells exposed to lead. Toxicology.

[36] Gidlow D.A. (2015). Lead toxicity. Occup. Med..

[37] Tsaih S.W., Korrick S., Schwartz J., Amarasiriwardena C., Aro A., Sparrow D., Hu H. (2004). Lead, diabetes, hypertension, and renal function: The normative aging study. Environ. Health Perspect..

[38] Harari F., Sallsten G., Christensson A., Petkovic M., Hedblad B., Forsgard N., Melander O., Nilsson P.M., Borné Y., Engström G. (2018). Blood Lead Levels and Decreased Kidney Function in a Population-Based Cohort. Am. J. Kidney Dis..

[39] Denic A., Glassock R.J., Rule A.D. (2016). Structural and functional changes within the aging kidney. Adv. Chronic Kidney Dis..

[40] Wiggins J.E., Patel S.R., Shedden K.A., Goyal M., Wharram B.L., Martini S., Kretzler M., Wiggins R.C. (2010). NFkappaB promotes inflammation, coagulation, and fibrosis in the aging glomerulus. J. Am. Soc. Nephrol..

[41] Houtkooper R.H., Pirinen E., Auwerx J. (2012). Sirtuins as regulators of metabolism and healthspan. Nat. Rev. Mol. Cell Biol..

[42] Tinkov A.A., Nguyen T.T., Santamaria A., Bowman A.B., Djordjevic A.B., Paoliello M.M.B., Skalny A.V., Aschner M. (2021). Sirtuins as molecular targets, mediators, and protective agents in metal-induced toxicity. Arch. Toxicol..

[43] Weinstein J.R., Anderson S. (2010). The aging kidney: Physiological changes. Adv. Chronic Kidney Dis..

[44] Karam Z., Tuazon J. (2013). Anatomic and physiologic changes of the aging kidney. Clin. Geriatr. Med..

[45] Rule A.D., Cornell L.D., Poggio E.D. (2011). Senile nephrosclerosis—Does it explain the decline in glomerular filtration rate with aging?. Nephron Physiol..

[46] Lerma E.V. (2009). Anatomic and physiologic changes of the aging kidney. Clin. Geriatr. Med..

[47] Pecly I.M., Genelhu V., Francischetti E.A. (2006). Renal functional reserve in obesity hypertension. Int. J. Clin. Pract..

[48] Abdelhafiz A.H., Brown S.H., Bello A., El Nahas M. (2010). Chronic kidney disease in older people: Physiology, pathology or both?. Nephron Clin. Pract..

[49] Nwankwo T., Yoon S.S., Burt V., Gu Q. (2013). Hypertension among adults in the United States: National Health and Nutrition Examination Survey, 2011–2012. NCHS Data Brief.

[50] Ruge T., Carlsson A.C., Larsson T.E., Carrero J.J., Larsson A., Lind L., ÄrnlFv J. (2014). Endostatin level is associated with kidney injury in the elderly: Findings from two community-based cohorts. Am. J. Nephrol..

[51] Li H., Wallin M., Barregard L., Sallsten G., Lundh T., Ohlsson C., Andersson E.M. (2020). Smoking-induced risk of osteoporosis is partly mediated by cadmium from tobacco smoke: The MrOS Sweden Study. J. Bone Miner. Res..

[52] Clarkson T.W., Magos L. (2006). The toxicology of mercury and its chemical compounds. Crit. Rev. Toxicol..

[53] Aaseth J., Wallace D.R., Vejrup K., Alexander J. (2020). Methylmercury and developmental neurotoxicity: A global concern. Curr. Opin. Toxicol..

[54] Bjørklund G., Dadar M., Mutter J., Aaseth J. (2017). The toxicology of mercury: Current research and emerging trends. Environ. Res..

[55] Song Y., Lee C.K., Kim K.H., Lee J.T., Suh C., Kim S.Y., Kim J.H., Son B.C., Kim D.H., Lee S. (2016). Factors associated with total mercury concentrations in maternal blood, cord blood, and breast milk among pregnant women in Busan, Korea. Asia Pac. J. Clin. Nutr..

[56] Rajaee M., Sanchez B.N., Renne E.P., Basu N. (2015). An investigation of organic and inorganic mercury exposure and blood pressure in a small-scale gold mining community in Ghana. Int. J. Environ. Res. Public Health.

[57] Li Y., Zhang B., Yang L., Li H. (2013). Blood mercury concentration among residents of a historic mercury mine and possible effects on renal function: A cross-sectional study in southwestern China. Environ. Monit. Assess..

[58] Sommar J.N., Svensson M.K., Bjor B.M., Elmstahl S.I., Hallmans G., Lundh T., Schon S.M., Skerfving S., Bergdahl I.A. (2013). End-stage renal disease and low-level exposure to lead, cadmium and mercury: A population-based, prospective nested case-referent study in Sweden. Environ. Health.

[59] Kim N.H., Hyun Y.Y., Lee K.B., Chang Y., Ryu S., Oh K.H., Ahn C. (2015). Environmental heavy metal exposure and chronic kidney disease in the general population. J. Korean Med. Sci..

[60] Rooney J.P. (2007). The role of thiols, dithiols, nutritional factors and interacting ligands in the toxicology of mercury. Toxicology.

[61] Cannon V.T., Zalups R.K., Barfuss D.W. (2001). Amino acid transporters involved in luminal transport of mercuric conjugates of cysteine in rabbit proximal tubule. J. Pharmacol. Exp. Ther..

[62] Bridges C.C., Zalups R.K. (2006). System b0,+ and the transport of thiol-s-conjugates of methylmercury. J. Pharmacol. Exp. Ther..

[63] Sabolić I., Breljak D., Škarica M., Herak-Kramberger C.M. (2010). Role of metallothionein in cadmium traffic and toxicity in kidneys and other mammalian organs. Biometals.

[64] Berlin M., Zalups R.K., Fowler B.A., Nordber G.F., Fowler B.A., Nordberg M. (2015). Mercury. Handbook on the Toxicology of Metals, Specific Metals II.

[65] Bridges C.C., Joshee L., van den Heuvel J.J., Russel F.G., Zalups R.K. (2013). Glutathione status and the renal elimination of inorganic mercury in the Mrp2(^−/−^) mouse. PLoS ONE.

[66] Šalamon Š., Kramar B., Marolt T.P., Poljšak B., Milisav I. (2019). Medical and dietary uses of N-acetylcysteine. Antioxidants.

[67] Ng C.F., Schafer F.Q., Buettner G.R., Rodgers V.G.J. (2007). The rate of cellular hydrogen peroxide removal shows dependency on GSH: Mathematical insight into in vivo H_2_O_2_ and GPx concentrations. Free Radic. Res..

[68] Cao Y., Skaug M.A., Andersen O., Aaseth J. (2015). Chelation therapy in intoxications with mercury, lead and opper. J. Trace Elem. Med. Biol..

[69] Kornhauser C., Garcia-Ramirez J.R., Wrobel K., Pérez-Luque E.L., Garay-Sevilla M.E., Wrobel K. (2008). Serum selenium and glutathione peroxidase concentrations in type 2 diabetes mellitus patients. Prim. Care Diabetes.

[70] Olson G.E., Whitin J.C., Hill K.E., Winfrey V.P., Motley A.K., Austin L.M., Deal J., Cohen H.J., Burk R.F. (2010). Extracellular glutathione peroxidase (Gpx3) binds specifically to basement membranes of mouse renal cortex tubule cells. Am. J. Physiol. Ren. Physiol..

[71] Alehagen U., Aaseth J., Alexander J., Brismar K., Larsson A. (2020). Selenium and Coenzyme Q10 Supplementation Improves Renal Function in Elderly Deficient in Selenium: Observational Results and Results from a Subgroup Analysis of a Prospective Randomised Double-Blind Placebo-Controlled Trial. Nutrients.

[72] Bjørklund G., Aaseth J., Ajsuvakova O.P., Nikonorov A.A., Skalny A.V., Skalnaya M.G., Tinkov A.A. (2017). Molecular interaction between mercury and selenium in neurotoxicity. Coord. Chem. Rev..

[73] Kieliszek M., Błażejak S., Bzducha-Wróbel A., Kot A.M. (2019). Effect of selenium on growth and antioxidative system of yeast cells. Mol. Biol. Rep..

[74] Rayman M.P., Stranges S. (2013). Epidemiology of selenium and type 2 diabetes: Can we make sense of it?. Free Radic. Biol. Med..

[75] Iglesias P., Selgas R., Romero S., Díez J.J. (2013). Selenium and kidney disease. J. Nephrol..

[76] Pakfetrat M., Malekmakan L., Hasheminasab M. (2010). Diminished selenium levels in hemodialysis and continuous ambulatory peritoneal dialysis patients. Biol. Trace Elem. Res..

[77] Ruiz A.A., Jiménez E.M., Bermejo-Barrera P., Lozano R., Seijas V.M.E. (2020). Selenium and All-cause Mortality in End-Stage Renal Disease. Retrospective Observational Cohort Study. J. Ren. Nutr..

[78] Burk R.F., Hill K.E. (2015). Regulation of Selenium Metabolism and Transport. Annu. Rev. Nutr..

[79] Willnow T.E., Christ A. (2017). Endocytic receptor LRP2/megalin-of holoprosencephaly and renal Fanconi syndrome. Pflug. Arch..

[80] Negri A.L. (2006). Proximal tubule endocytic apparatus as the specific renal uptake mechanism for vitamin D-binding protein/25-(OH)D3 complex. Nephrology.

[81] Bjørklund G. (2015). Selenium as an antidote in the treatment of mercury intoxication. Biometals.

[82] Sugiura Y., Tamai Y., Tanaka H. (1978). Selenium Protection against Mercury Toxicity; High Binding Affinity of Methylmercury by Selenium-containing Ligands in Comparison with Sulfur-containing Ligands. Bioinorg. Chem..

[83] Kuria A., Fang X., Li M., Han H., He J., Aaseth J.O., Cao Y. (2020). Does dietary intake of selenium protect against cancer? A systematic review and meta-analysis of population-based prospective studies. Crit. Rev. Food Sci. Nutr..

[84] El-Khairy L., Ueland P.M., Refsum H., Graham I.M., Vollset S.E. (2001). Plasma total cysteine as a risk factor for vascular disease: The European Concerted Action Project. Circulation.

[85] Spiller H.A., Hays H.L., Casavant M.J. (2021). Rethinking treatment of mercury poisoning: The roles of selenium, acetylcysteine, and thiol chelators in the treatment of mercury poisoning: A narrative review. Toxicol. Commun..

